# Hill’s Soft Filling

**Published:** 1848-07

**Authors:** 


### HILL'S SOFT FILLING.

We see in the Dental Recorder an article under the above caption. We apprehend that very *soft* fillings will not answer a very good purpose. We have supposed, however, since the first notice of the Gutta Percha reached us, that a preparation of this—with lime in some of its forms—would be discovered, which would answer a valuable purpose in the Profession. Several years since, we instituted a series of experiments with the Caoutchouc and other substances, hoping to secure something which might answer the purpose of amalgam, and be free from its objections. We found, however, that the Caoutchouc, even in its etherial solution, possessed not enough hardness, when mixed with the lime, to answer a valuable purpose. Out of the mouth it would harden admirably; but the heat and moisture of the mouth kept it too soft for dental purposes. We presume that the Gutta Percha possesses more of the requisite qualities, and intend at our first leisure to test its properties. We know not if Hill's soft filling is thus prepared, yet we feell assured that a *soft* filling can thus be made, and intend, if possible, to thus make a *hard* one. CIN. ED.

